# Immunogenicity of HIV-1 Env-Gag VLP mRNA and Adenovirus Vector Vaccines in Mice

**DOI:** 10.3390/vaccines13121242

**Published:** 2025-12-14

**Authors:** Jing Yang, Qi Ma, Xiaozhou He, Hongxia Li, Xiaoguang Zhang, Yanzhe Hao, Xia Feng

**Affiliations:** National Key Laboratory of Intelligent Tracking and Forecasting for Infectious Diseases, National Institute for Viral Disease Control and Prevention, Chinese Center for Disease Control and Prevention, Beijing 100052, China; yangjing@ivdc.chinacdc.cn (J.Y.); 13179818951@163.com (Q.M.); hexz@ivdc.chinacdc.cn (X.H.); lihx@ivdc.chinacdc.cn (H.L.); zhangxg@ivdc.chinacdc.cn (X.Z.)

**Keywords:** human immunodeficiency virus, messenger RNA vaccine, virus-like particle vaccine, adenovirus vector vaccines

## Abstract

Background/Objectives: In previous studies, we demonstrated that the HIV-1 Env-Gag VLP mRNA vaccine elicited superior cellular immune responses. In this study, we further evaluated the immunogenicity of the Env-Gag VLP mRNA and adenovirus vector vaccines when administered individually or in combination in mice. Methods: BALB/c mice were divided into four groups and immunized twice at a 3-week interval. The three groups received either the Env-Gag VLP mRNA vaccine, the adenovirus vector vaccines expressing env and gag genes, or PBS as a control. The fourth group received a prime-boost regimen, primed with the Env-Gag mRNA vaccine and boosted with the adenovirus vector vaccines. The HIV-1 specific cellular and humoral immune responses were measured 1, 2, 4 and 8 weeks after the last immunization. Results/Conclusions: The results showed that the mRNA vaccines prime-adenovirus vector vaccines boost elicited higher cellular immune responses than those induced by homologous regimens at multiple time points, especially 8 weeks after the last immunization. Although the level of gp120 binding antibody in the combined immunization group is significantly lower than that of in the VLP mRNA vaccine group, a more balanced Th1/Th2 responses were induced in the combined immunization group, and significantly higher and longer-lasting neutralizing antibody levels were detected in this group making it a very promising HIV vaccine strategy.

## 1. Introduction

The prime-boost immunization strategy has been widely applied in the development of vaccines targeting various infectious diseases [1,2]. A well-established example of this approach is the use of a DNA vaccine for priming, followed by a booster with a different vaccine type, most commonly a viral vector vaccine [3]. Such heterologous prime–boost regimens have shown strong immunogenicity in the development of numerous vaccines, including candidates against HIV [4,5,6,7,8,9]. Their major advantage is the synergistic effect between different vectors, which can markedly enhance the overall immune response. In addition, pre-existing immunity to a given vector may compromise antigen-specific responses and thereby reduce protective efficacy; combining different vectors can help alleviate this limitation [10,11].

Researchers tend to use lower-seroprevalence vectors, such as Ad26, Ad35, or simian/chimpanzee adenovirus, in prime-boost regimens due to the complex interactions between adenovirus vector and pre-existing vector-specific immune responses [12]. Virus-like particles (VLPs) are self-assembled, nanoscale structures composed of one or more viral structural proteins that mimic the morphology and antigenic organization of native virions but lack viral genetic material and are therefore non-infectious and replication-deficient [13,14,15]. Several VLP-based vaccines, such as those targeting hepatitis B virus and human papillomavirus, have been successfully licensed for human use, demonstrating excellent safety profiles and durable protection, and a growing number of VLP platforms are being developed against emerging infectious diseases and cancers [16,17].

The virus-like particle (VLP) vaccine platform offers several advantages [18,19]. These self-assembled, non-infectious particles mimic authentic viruses by displaying antigens in a dense, repetitive pattern on their surfaces. Their repetitive structure facilitates extensive cross-linking with B cell receptors, leading to the potent induction of humoral immunity [20,21]. Enhancing humoral immunity through VLPs has thus been a key strategy in vaccine development in recent years [22,23].

During the development of HIV-1 vaccines, the heterologous “prime-boost” strategy has been proven effective. Recombinant envelope (Env) glycoproteins can induce neutralizing antibody responses specific to certain viral isolates, as well as cytotoxic T lymphocyte (CTL) responses. Vaccines targeting the Gag core protein as an antigen can elicit robust and specific cellular immune responses but fail to generate protective antibodies. Co-expressing these two functionally distinct antigens can lead to the formation of virus-like particles (VLPs). The formation of VLPs in our system is primarily driven by the HIV-1 Gag protein, which has an inherent ability to self-assemble into the structural core of a particle upon expression in cells. HIV-1 Env protein is a transmembrane glycoprotein that is expressed on the surface of host cells infected by HIV-1 and on the surface of mature HIV-1 virions. By co-expressing env and gag mRNA, we generated VLPs that mimic the natural HIV infection process, thereby enabling the dual presentation of Env proteins on the cell surface and VLPs. This strategy enhances B cell activation and stimulates higher antibody titers. This approach not only compensates for the individual limitations of each antigen but may also achieve better overall protective efficacy compared to using either immunogen alone [24].

In previous studies, we developed a VLP mRNA vaccine co-expressing HIV-1 Env and Gag proteins that induced potent humoral and cellular immune responses in mice. In this study, we evaluated this VLP mRNA vaccine alone and combined with adenovirus vector vaccines expressing the same Env and Gag proteins in mice. The heterologous “mRNA prime-Adenovirus boost” strategy outperformed the homologous mRNA or Adenovirus regimen by eliciting a balanced Th1/Th2 response and inducing higher, more persistent neutralizing antibody levels. Further investigation is justified.

## 2. Materials and Methods

### 2.1. HIV-1 Env-Gag VLP mRNA and Adenovirus Vector Vaccines

The mRNA and AdV vaccines used in this study were described in the previous literature [25,26,27]. We constructed two LNP-encapsulated, nucleoside-modified mRNA vaccines encoding a truncated HIV-1 CRF01_AE Env (gp145) and a full-length HIV-1 subtype B Gag protein. The vaccines exhibited uniform nanoparticle size (~110 nm). A recombinant adenovirus vector vaccine Ad5F35-AEgp145, which expresses the HIV-1 CRF01_AE Env (gp145) antigen, was also constructed. Electron microscopic analysis demonstrated that the vaccine particles exhibited a typical adenoviral morphology with an approximate diameter of 70 nm. The recombinant adenovirus serotype 5 vaccine (Ad5-gag) was constructed to express a codon-optimized HIV-1 subtype B’ Gag protein.

### 2.2. Mouse Immunization and Detection Protocols

**Figure 1:**
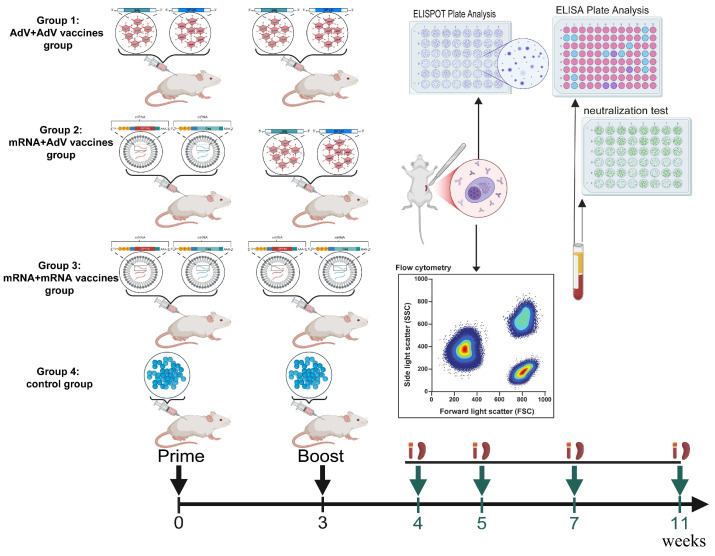
Schedule of Immunization and detection strategies in Balb/c mice.

The experimental design for mouse immunization is outlined below. The mice were divided into four groups, each consisting of 20 mice. The groups established were adenovirus vector vaccine alone immunization group (AdV + AdV), mRNA vaccine alone immunization group (mRNA + mRNA), primed with the mRNA vaccine and boosted with the adenovirus vector vaccine group (mRNA + AdV), and the PBS control group. The immunization dosage for the adenovirus vector vaccine is 10^7^ PFU Ad5F35-AEgp145 plus 107 PFU Ad5-HIVgag per 100 μL per mouse. The mRNA vaccine immunization dosage is 2.5 μg AEgp145-mRNA plus 2.5 μg gag-mRNA per 100 μL per mouse. The PBS immunization dosage is 100 μL per mouse. The injections are administered into the tibialis anterior muscle of the mice at a single site. The specific immunization and testing schedule is shown in Figure 1. At 1 week, 2 weeks, 4 weeks, and 8 weeks after the final immunization, five mice from each group were selected. First, their body weight was monitored. Then, the mice were anesthetized via intraperitoneal injection of 20 µg sodium pentobarbital per gram of body weight. Blood was collected from the orbital cavity to separate serum for assessing the humoral immune response. Spleens were harvested to isolate splenic lymphocytes for evaluating the cellular immune response.

### 2.3. Enzyme-Linked Immunosorbent Assay (ELISA)

Indirect ELISA was used to assess the levels of HIV-1 Gp120-specific IgG, IgG1, and IgG2a binding antibodies, as well as Ad5F35 conjugated antibodies, in sera from immunized mice. Briefly, purified HIV-1/Clade AE Gp120 protein (100 ng/well) or Ad5F35 (8 × 10^8^ VP/well) was diluted to working concentration using coating buffer and added to 96-well plates at 100 µL per well, followed by overnight coating at 4 °C. After discarding the coating solution, plates were blocked with 5% skim milk in PBS at 37 °C for 2 h. Serum samples were serially diluted, added to the wells, and incubated at 37 °C for 1 h. After washing five times with PBST, horseradish peroxidase (HRP)-conjugated goat anti-mouse IgG, IgG1, or IgG2a secondary antibodies (Abcam, Cambridge, MA, USA; diluted 1:20,000) were added and incubated at 37 °C for 1 h. Following another wash step, TMB substrate solution (Wan Tai, Beijing, China) was added to each well and incubated at 37 °C for 30 min for color development. The reaction was stopped using TMB stop solution (Beyotime, Beijing, China). Absorbance was measured at 450 nm with a reference wavelength of 630 nm using a microplate reader (BioRad, Hercules, CA, USA). The endpoint antibody titer was defined as the highest serum dilution at which the absorbance value exceeded twice the background signal.

### 2.4. Pseudovirus-Based Neutralization Assay

Mouse serum was inactivated by heat treatment at 56 °C for 30 min to eliminate complement activity. A total of 20 TCID_50_ of HIV-1 AE pseudovirus was mixed with serially two-fold diluted heat-inactivated serum (starting at a dilution of 1:10), in the presence of DEAE-dextran at a final concentration of 40 µg/mL. The virus–serum mixture was incubated at 37 °C for 1 h and then added to pre-seeded TZM-bl cells (6 × 10^3^ cells per well). After 48 h of incubation at 37 °C, cells were lysed and luciferase activity was measured. All assays were performed in duplicate. All assays were performed in duplicate. The virus-only control (without serum) was set as 100% luciferase activity, and neutralization activity of each serum-virus dilution was calculated as the percentage reduction in luciferase activity relative to the control. The resulting data were analyzed using the Excel spreadsheet template provided in the protocol “Preparation and Titration of HIV-1 Envelope Pseudovirus” (released by the Montefiori Laboratory at Duke University, Durham, NC, USA in October 2021) to determine the TCID_50_ of the HIV pseudovirus.

### 2.5. Enzyme Linked Immunospot (ELISPOT) Assay

To assess the frequency of Env/Gag-specific IFN-γ-secreting cells in mouse splenic lymphocytes, an IFN-γ ELISPOT assay kit (Mabtech AB, Nacka, Sweden) was used according to the manufacturer’s instructions. Briefly, splenic lymphocytes were isolated from mouse spleens using mouse lymphocyte separation medium (Dakewe, Beijing, China). Freshly isolated lymphocytes were plated at 2 × 10^5^ cells per well in replicate and stimulated with 2 µg/mL of an H-2d-restricted Env+Gag-specific cytotoxic T-lymphocyte (CTL) epitope peptide. Cells were seeded into 96-well plates pre-coated with anti-mouse IFN-γ monoclonal antibody and incubated for 48 h at 37 °C with 5% CO_2_. Background control wells contained cells cultured in 1% DMSO (Sigma, St. Louis, MO, USA), while positive control wells were stimulated with 25 ng/mL phorbol 12-myristate 13-acetate (PMA; Sigma, USA) plus 1 µg/mL ionomycin (Sigma, USA). After incubation, spots were developed following the kit protocol. Plates were air-dried, and spots were quantified using an ImmunoSpot reader (CTL, Cleveland, OH, USA). A response was considered positive when the peptide-stimulated wells showed at least a four-fold increase in spot-forming cells (SFCs) compared with the control, and the number of SFCs exceeded 50 per 10^6^ splenic lymphocytes.

### 2.6. Intracellular Cytokine Staining (ICS) by Flow Cytometry

1 × 10^6^ splenocytes were incubated with a final concentration of 2 μg/mL peptide stimulants, while mock negative controls received an equivalent volume of medium containing DMSO and positive controls were incubated with a final concentration of 25 ng/mL of PMA and 1 µg/mL of ionomycin. Monensin Solution (Cat.No.505808, biolegend, San Diego, CA, USA) was added and incubated for 6 h to block cytokine release. The cells were then harvested and washed with PBS. The cells were subsequently stained with Zombie aqua (Cat.No.423102, BioLegend) for 10 min to identify live and dead cells. Samples were washed twice to remove Zombie aqua, after incubation with TruStain FcX™ (Cat.No.101319, biolegend), the splenocytes were stained with FITC anti-mouse CD3 antibody (Cat.No.100204, biolegend), PerCP/Cyanine5.5 anti-mouse CD4 antibody (Cat.No.100540, biolegend), and APC/Cyanine7 anti-mouse CD8a antibody (Cat.No.100714, biolegend), for 30 min at 4 °C followed by cell fixation and permeabilization (Cat.No.420801, Cat.No.421002, biolegend). Subsequently, the cells were stained with PE anti-mouse IFN-γ (Cat.No.505808, biolegend), Brilliant Violet 421 anti-mouse IL-2 (Cat.No.503826, biolegend) for 30 min at room temperature. After washing with permeabilization wash buffer, the multifunctional T cells co-expressing IFN-γ and IL-2 were analyzed using flow cytometry on a BD FACS Aria II flow cytometer.

### 2.7. Data Analysis

The humoral immune level is represented by the geometric mean of titers of HIV-1 gp120-specific binding antibodies, Ad5F35 conjugated antibodies, and neutralizing antibodies in mouse serum. The specific cellular immune response level detected by the method is expressed as the average number of HIV Env/Gag-specific IFN-γ spot-forming cells (SFCs/10^6^ splenocytes) per million splenic lymphocytes. Statistical analysis was performed using GraphPad Prism 7.0 software (version 7.0; GraphPad Software, La Jolla, CA, USA), applying two-way ANOVA to compare the intensity of immune responses among different groups of mice at various time points, with differences considered statistically significant at *p* < 0.05. For comparisons involving multiple groups and time points, data were analyzed by two-way ANOVA followed by Sidak’s multiple-comparison test; *p* < 0.05 was considered statistically significant.

## 3. Results

### 3.1. Immune Response Types Triggered by Homologous and Heterologous Immunization with mRNA and Adenovirus Vector Vaccine

**Figure 2:**
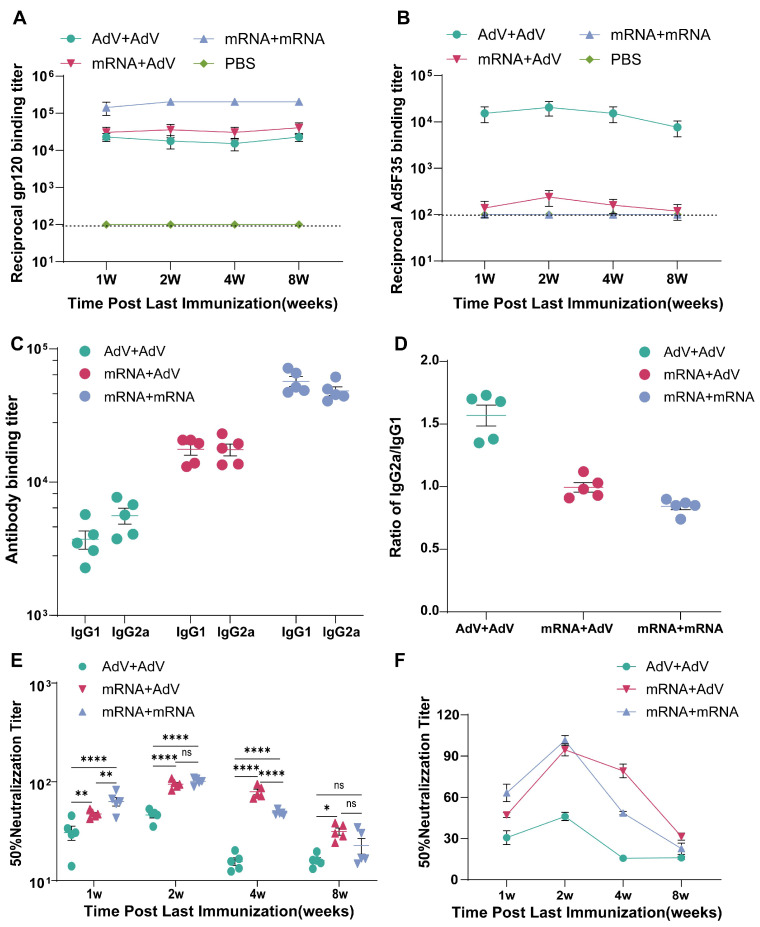
Humoral immune responses in mice sera. (**A**) The changes in the levels of serum HIV-1 gp120 binding antibodies in mice after immunization with different vaccines over time. (**B**) The changes in the levels of adenovirus Ad5F35-binding antibodies in mice after immunization with different vaccines over time. (**C**) The levels of specific IgG1 and IgG2a antibodies in the sera of immunized mice. (**D**) the ratio of IgG2a/IgG1 in the sera of immunized mice. (**E**) The level of neutralizing antibody in sera of immunized mice post last immunization. (**F**) The changes in the level of neutralizing antibody in sera of immunized mice post last immunization. * *p* < 0.05; ** *p* < 0.01; **** *p* < 0.0001; ns, no significant difference.

In order to determine a superior immunization strategy for mRNA vaccine and Adenovirus vector vaccine, our synthesized mRNA vaccine (mRNA) and Adenovirus vector vaccine (AdV) were used for homologous and heterologous prime-boost immunization. BALB/c mice were immunized twice with different immunization strategies at 3-week intervals. Serum samples were collected at 1 week, 2 weeks, 4 weeks and 8 weeks after the last immunization, and gp120-specific binding antibody levels were determined by indirect ELISA. The results showed that one week after the last immunization, all vaccine groups produced high-titer gp120 binding antibodies (Figure 2A). Among them, the gp120 binding antibody titers of mice in the AdV + AdV group and the mRNA + AdV group that received combined immunization were comparable. However, the gp120 binding antibody titer of mice in the mRNA + mRNA vaccine group that received single immunization was significantly higher than those of the above two experimental groups, reaching 10^6^. During the subsequent experimental period, the total IgG levels of all groups remained stable.

We also evaluated the specific IgG1 and IgG2a antibody titers, as well as the IgG2a/IgG1 ratio in the serum of mice four weeks after the final immunization (Figure 2C,D). The AdV + AdV group alone induced a higher IgG2a antibody titer against gp120, with an IgG2a/IgG1 ratio of 1.568, suggesting that the adenovirus vector vaccine induced a Th1-biased immune response [28]. Mice vaccinated with the mRNA + mRNA vaccine alone exhibited higher IgG1 antibody titers against gp120, with an IgG2a/IgG1 ratio of 0.842, indicating that the mRNA vaccine induced a Th2-biased immune response. Mice primed with the mRNA vaccine and boosted with the adenovirus vector vaccine showed elevated titers of both IgG1 and IgG2a antibodies against gp120, with an IgG2a/IgG1 ratio of 0.994, suggesting that the combined immunization strategy induced a balanced Th1/Th2 response.

### 3.2. The Levels of Adenovirus Ad5F35-Binding Antibodies in Mice Following Vaccination

The adenovirus Ad5F35 binding antibody titers in mice after vaccine immunization are shown in Figure 2B. Within 8 weeks after the final immunization, the serum Ad5F35 binding antibody levels in the mRNA + mRNA vaccine group were comparable to those in the group that received only PBS, remaining at baseline levels (1:100). Throughout the experiment, the serum Ad5F35 binding antibody levels in the AdV + AdV vaccine group were significantly higher than those in other immunization groups. One week after the final immunization, the serum Ad5F35 binding antibody titer in the mRNA + AdV vaccine group was relatively low, at 1:140, while the AdV + AdV vaccine group had a titer level of 1:15,360. Two weeks after the final immunization, the serum Ad5F35 binding antibody titer level in the mRNA + AdV vaccine group reached its peak at 1:240, and the AdV + AdV vaccine group also reached its peak at 1:20,480. Four weeks after the final immunization, the serum Ad5F35 binding antibody titers in both the AdV + AdV vaccine group and the mRNA + AdV vaccine group began to decline to 1:15,360 and 1:160, respectively. Eight weeks after the final immunization, the serum Ad5F35 binding antibody titers in both groups dropped to their lowest levels, at 1:7680 and 1:120, respectively.

### 3.3. Detection of HIV-1 Subtype AE-Specific Neutralizing Antibodies in Immunized Mouse Serum

The neutralizing activity of the mouse serum was tested using a pseudovirus-based neutralization assay (Figure 2E). The initial dilution of the tested mouse sera was 1:10, HIV-1 AE specific neutralizing antibodies were induced in all vaccine immunization groups. One week after the final immunization, the mRNA + mRNA vaccine group exhibited the highest level of neutralizing antibodies, with a 50% neutralizing antibody titer of 1:63.2. The mRNA + AdV vaccine group followed, with a titer of 1:47.2. The lowest neutralizing antibody level was observed in the AdV + AdV vaccine group, with a titer of 1:30.6. Two weeks post-final immunization, the serum neutralizing antibody titers in all vaccine groups reached their peak. the mRNA + mRNA vaccine group and the mRNA + AdV vaccine group showed comparable neutralizing antibody levels, with 50% titers of 1:101.4 and 1:94.6, respectively. The AdV + AdV vaccine group remained the lowest, with a titer of 1:46.1. Four weeks after the final immunization, the neutralizing antibody titers in the serum of mice began to decline across all vaccine groups. The titers in the AdV + AdV vaccine group and the mRNA + mRNA vaccine group decreased more rapidly, with 50% titers of 1:15.9 and 1:48.6, respectively. In contrast, the decline in the mRNA + AdV vaccine group was slower, with a titer of 1:79.3. Eight weeks post-final immunization, the 50% neutralizing antibody titer in mRNA + AdV vaccine group dropped to 1:31.4, slightly higher than the mRNA + mRNA vaccine group, which had a titer of 1:22.6; however, the difference was not statistically significant. The AdV + AdV vaccine group maintained a titer comparable to that observed at four weeks post-final immunization, remaining at 1:15.9 (Figure 2F).

### 3.4. Detection of Cellular Immune Responses in Mice After Immunization

**Figure 3:**
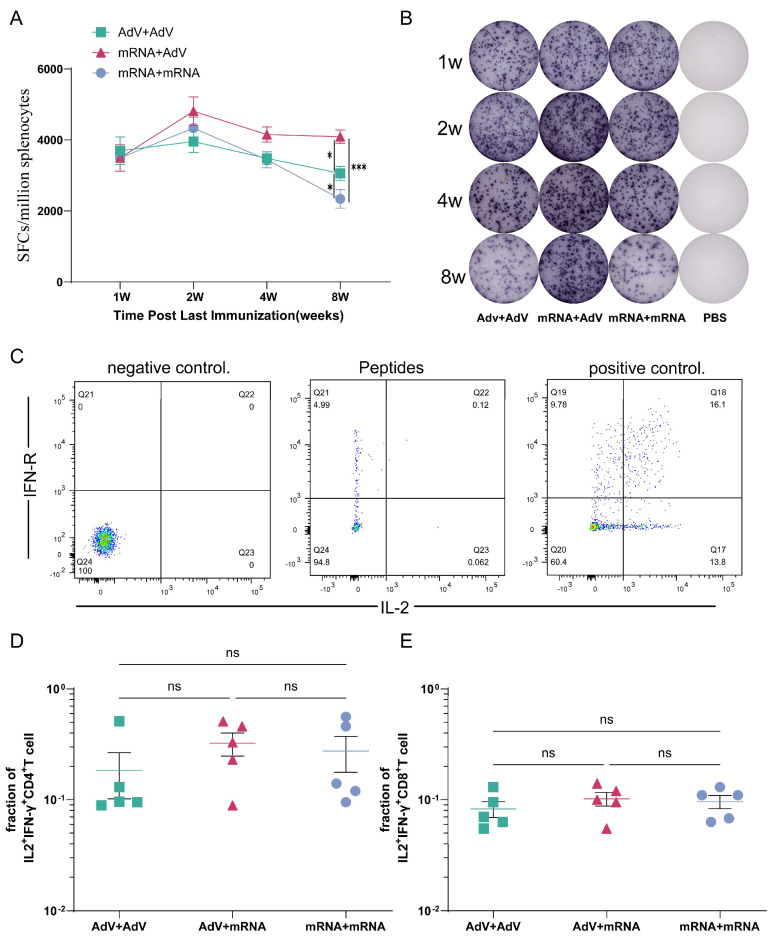
Cellular immune responses elicited by different vaccines in mice. (**A**,**B**) The levels of secreted IFN−γ in spleen lymphocytes were measured by the ELISPOT assay (SFCs is an abbreviation for “Spots Forming Cells”). (**C**) A graphical representation of the ICS result. (**D**,**E**) Frequency of multifunctional T cells co-expressing IFN−γ and IL−2 detected by flow cytometry in both CD8^+^ and CD4^+^ T cells. * *p* < 0.05; *** *p* < 0.001; ns, no significant difference.

As shown in Figure 3A, one week after the final immunization, HIV-1 AEgp145-specific cellular immune responses were detectable in all experimental groups except for the control group, with no statistically significant differences in immune response levels between the groups. Two weeks post-immunization, specific cellular immune responses in all experimental groups peaked, with the mRNA + AdV vaccine group reaching the highest level of 4809 SFCs/10^6^ splenocytes, followed by the mRNA + mRNA vaccine group at 4335 SFCs/10^6^ splenocytes. The AdV + AdV vaccine group showed the lowest immune response at 3955 SFCs/10^6^ splenocytes, but again, no statistically significant differences in immune response levels were observed between the groups. Four weeks after the final immunization, the specific cellular immune responses in all experimental groups declined, with the mRNA + AdV vaccine group maintaining the highest level of 4153 SFCs/10^6^ splenocytes. The mRNA + mRNA vaccine group and the AdV + AdV vaccine group both decreased to the same level, with values of 3483 SFCs/10^6^ splenocytes and 3441 SFCs/10^6^ splenocytes, respectively. Eight weeks after the final immunization, the mRNA + AdV vaccine group still maintained 4093 SFCs/10^6^ splenocytes, while the AdV + AdV vaccine group slightly decreased to 3059 SFCs/10^6^ splenocytes, and the mRNA + mRNA vaccine group decreased to 2339 SFCs/10^6^ splenocytes. We also evaluated the T cell response following the booster immunization.

IL-2 and IFN-γ are primarily produced by activated T cells, and their secretion levels reflect the type and status of T-cell immune responses. At one week after the final immunization, multifunctional T cells co-expressing IFN-γ and IL-2 were detected by flow cytometry in both CD8^+^ and CD4^+^ T cells. The results showed no significant differences in immune responses induced by the three vaccine combinations (Figure 3D,E).

### 3.5. Safety Testing of Mice After Vaccine Immunization

**Figure 4:**
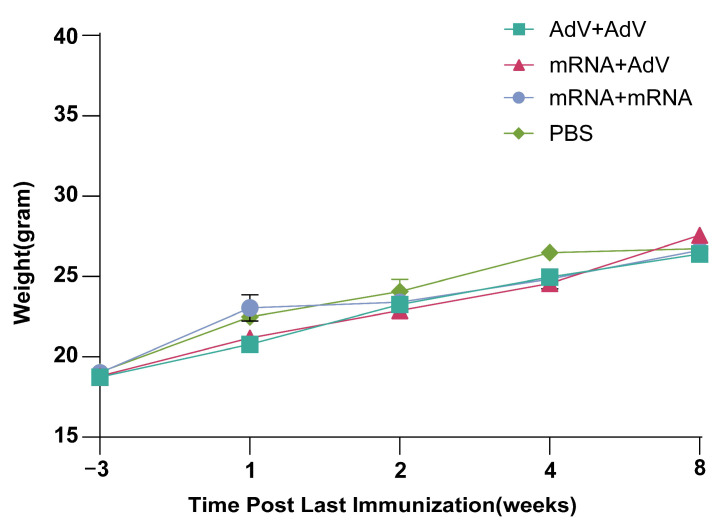
Changes in body weight of mice after vaccination.

Body weight of the mice was monitored at one, two, four, and eight weeks following the final vaccination. As shown in Figure 4, no significant differences in body weight were observed between the vaccine-immunized group and the PBS-immunized control group. Furthermore, there were no notable variations in body weight across the groups over time.

## 4. Discussion

In this study, we conducted a comprehensive evaluation of the immune effects of adenovirus vector and mRNA vaccines in mice, with a particular emphasis on their performance in a heterologous prime-boost immunization strategy. This immunization approach combines the characteristics of both vaccine platforms to enhance overall immune efficacy. Our results indicate that the cellular immune responses induced by the heterologous regimen were higher at multiple time points compared to groups immunized with either vaccine alone, with the most significant difference observed at 8 weeks post the final immunization. Although the levels of gp120-binding antibody in the combined immunization group were significantly lower than those in the mRNA vaccine-alone group, the heterologous strategy elicited a balanced Th1/Th2 immune response. furthermore, the peak levels of HIV-1 subtype AE-specific neutralizing antibodies induced by the combined immunization were comparable to those induced by the mRNA vaccine alone, and the decline in neutralizing antibody levels was slower in the combined immunization group. In immune experiments, the observed decrease in binding antibody titers may not contradict the persistence of neutralizing antibodies. The dissociation between binding and neutralizing antibodies has been previously reported. Riou et al. [29] found that a booster dose of the full-dose Ad26 vector vaccine did not significantly increase binding antibody levels, yet it still led to a modest enhancement in neutralizing antibodies—approximately 2.1-fold. But its underlying reasons require further investigation. In contrast, the significant decline in neutralizing antibodies over time following homologous mRNA vaccination has been well-documented in numerous studies [30].

Adenovirus vector vaccines are generally effective at activating a robust cellular immune response, particularly a CD8^+^ T cell response, which represents a major advantage [31,32]. The single-dose immunization results from this study demonstrate that the vaccine can rapidly induce a cellular immune response with good persistence. However, the application of virus vector-based vaccines has certain limitations. The primary constraint is that after the initial immunization, the body may develop an immune response against the vector itself. Upon subsequent administration, pre-existing vector-specific antibodies quickly recognize and bind to the vaccine, potentially reducing the expression level of the antigen gene and thereby compromising vaccine efficacy. According to previous studies, the prevalence of type 5 adenovirus infection in China is relatively high, with over 77% of the population testing positive for adenovirus-neutralizing antibody. This is one of the factors that limiting the widespread use of adenovirus vector vaccines [33,34]. In our study, mice that received two doses of the adenovirus vector vaccines generated Ad5F35-binding antibodies at titers of approximately 1:20,480, whereas mice immunized with only one dose of the adenovirus vector vaccine (in the mRNA-primed, adenovirus vector-boosted group) generated relatively low levels of Ad5F35-binding antibodies, with titers ranging from 1:120 to 1:240. The heterologous prime-boost immunization strategy demonstrated better immune efficacy than the homologous vector boosting strategy, without eliciting the strong anti-vector immune response observed after two doses of the viral vector vaccine. Our data are consistent with the findings of previous studies [35,36], indicating that the presence of neutralizing antibodies against the vector (e.g., adenovirus) and memory immune cells significantly reduces both the antigen expression level and the intensity of immune responses elicited by subsequent homologous booster doses. Switching to a different vaccine platform can effectively circumvent pre-existing immunity against the vector, thereby more efficiently re-stimulating the immune system against the target pathogen.

A well-designed heterologous prime-boost strategy can broaden the breath of immune responses. In one study, mice primed with a DNA vaccine carrying ESAT6 and boosted with the same antigen in the form of a recombinant protein showed a significant increase in the secretion of Th1-type cytokines, along with a notable rise in the IgG2/IgG1 ratio [37]. In another investigation, mice were primed with a DNA vaccine expressing the HSV-2 gD antigen, which predominantly induces a Th1-type cellular immune response. This was followed by a booster immunization with recombinant gD protein (which mainly induces a Th2-polarized response). The results indicated that this combined immunization strategy significantly enhanced antibody levels, T cell proliferation capacity, and the production of Th1-type cytokines [38]. In our experiment, a similar phenomenon was observed. Compared to immunization using only homologous vector vaccines, mice that were primed with an mRNA vaccine and boosted with an adenovirus vector vaccine exhibited higher titers of anti-gp120 IgG1 and anti-gp120 IgG2a antibodies. The IgG2a/IgG1 ratio indicated that this immunization strategy elicited a balanced Th1/Th2 response in Balb/c mice.

The prime-boost vaccination strategy can further enhance the efficacy of existing vaccines. For instance, in animal experiments, an initial immunization with a DNA vaccine followed by a booster with an inactivated rabies vaccine resulted in a superior immune response. DNA priming can enhance the potency and persistence of high-titer immune sera produced in animals immunized with recombinant PA antigen to protect against anthrax [39]. After initial immunization with DNA, mice boosted with the hepatitis B surface protein vaccine exhibited a stronger and more uniform antibody response compared to the group receiving only recombinant protein. Additionally, higher levels of IL-12 and IFN-γ secretion were observed in splenocytes [40]. In our study, compared to administering only an adenovirus vector vaccine or an mRNA vaccine, priming with an mRNA vaccine followed by boosting with an adenovirus vector vaccine resulted in a longer-lasting and higher peak specific cellular immune response, as well as more sustained levels of HIV-1 AE-specific neutralizing antibodies. This suggests that a heterologous prime-boost immunization strategy can achieve better immune outcomes.

In terms of biosafety, there was no difference in body weight between the vaccine-immunized and control group of mice over the time. The homologous and heterologous immunization groups did not show a significant increase in adverse reactions compared to the control group, supporting the safety of this strategy. Additionally, a key feature of combined immunization is the persistence of neutralizing antibodies, which may be related to the vaccine’s ability to stimulate a diverse population of immune cells, thereby helping to maintain long-term pathogen defense capabilities. Future research should focus on further optimizing immunization protocols with different doses and time intervals and explore the application of heterologous prime-boost strategies in other animal models and humans, especially for achieving long-term protective efficacy against complex pathogen infections.

## 5. Conclusions

In summary, the combined use of adenovirus vector vaccine and mRNA vaccines has demonstrated promising results in animal models, offering new insights and potential for broader applications. As research progresses, this combination strategy is expected to play an important role in vaccine development and pandemic control. Further studies should be conducted to confirm its advantages in long-term immune memory and sustained protection.

## Data Availability

All data related to this study are included in this article.
